# Indigenous Knowledge: Revitalizing Everlasting Relationships between Alaska Natives and Sled Dogs to Promote Holistic Wellbeing

**DOI:** 10.3390/ijerph20010244

**Published:** 2022-12-23

**Authors:** Janessa Newman, Inna Rivkin, Cathy Brooks, Kathy Turco, Joseph Bifelt, Laura Ekada, Jacques Philip

**Affiliations:** 1 Tamamta Fellowship, University of Alaska Fairbanks, Fairbanks, AK 99775, USA; 2 Department of Psychology and Center for Alaska Native Health Research, University of Alaska Fairbanks, Fairbanks, AK 99775, USA; 3 Department of Alaska Native Studies and Rural Development, University of Alaska Fairbanks, Fairbanks, AK 99775, USA; 4 Alaska’s Spirit Speaks: Sound & Science, Fairbanks, AK 99709, USA; 5 A-CHILL (Alaska Care and Husbandry Instruction for Lifelong Learning) Project, Tok, AK 99780, USA; 6 Rampart School (Yukon-Koyukuk School District), Rampart, AK 99767, USA; 7 BLaST (Biomedical Learning and Student Training) Program, University of Alaska Fairbanks, Fairbanks, AK 99775, USA; 8 Center for Alaska Native Health Research, University of Alaska Fairbanks, Fairbanks, AK 99775, USA

**Keywords:** Indigenous peoples, Alaska Native youths, digital storytelling, connection to land, wellbeing, community-based participatory research, Indigenous knowledge, rural, intergenerational mentorship

## Abstract

Introduction: Indigenous peoples have documented their culture’s history in oral stories, revealing lessons about holistic relationships fostering perseverance. Despite vast differences in time, relationships and stories are equally important today. Athabascans retain their values, life skills, and wellness through cultural practices. Creating opportunities for youths to learn through reciprocal relationships increases wellness in Indigenous communities, highlighting the significance of community-developed programs that connect youths to their place and culture. Method: Athabascan youths in rural Alaska get hands-on experience and Elder mentorship working with sled dogs in the Frank Attla Youth and Sled Dog Care-Mushing Program (FAYSDP). Through a community-based participatory research partnership with the community of Huslia and Jimmy Huntington School, we examined how FAYSDP affects youths, and how relationships within culture and land promote wellbeing. Fifteen middle and high-school youths shared their perspectives on how FAYSDP impacts them and their community using photovoice and digital storytelling. Nineteen adults contributed their perspectives in focus groups. We used emerging themes (inductive) and Athabascan cultural values and elements of social capital (deductive approaches) to analyze the qualitative data. Results: Findings illustrate how FAYSDP promotes wellbeing by empowering youths to apply what they learn to generate their own knowledge, while centering communities around culturally significant practices such as dog mushing. It connects youths to their home and their cultural values, using dogs as the driving force to bridge generations and foster youth wellness. Discussion: We discuss implications for community-based programs that engage Elders as teachers and the land as their classroom to promote youth holistic wellness.

## 1. Introduction

### 1.1. A Story of Holistic Connection

The following story captures a shared real experience by two Indigenous scholars living in an Alaska village with their sled dogs—me, lead author Janessa, and my partner, Joe. Our story is told in tandem, as we each experienced the same event from different perspectives. My reflections are in regular font; Joe’s are in italics.

Once a vibrant dog mushing community, the Koyukon Athabascan village of Rampart had not had sled dogs in several decades, and seeing a dog team running down the old trails brought pride to the small community. By running dogs, Joe is continuing the legacy of his grandfather, who came from Huslia, another Koyukon Athabascan village.

One winter evening, the sun was setting beyond the horizon. Joe and I were preparing to take our sled dogs on a training run. The temperature hovered around a sluggish −15 °F (−26 °C), and the stress of modern life’s obligations weighed heavy on our minds. *It was 15 February, the day my grandpa, George Attla Jr., passed away 7 years ago. At times, I would yearn for my grandpa, his presence, his advice, and wonder if he is proud of the work I am doing with the youths. During those times, there always seemed to be an almost intuitive sign that eased my mind; often, the sign came in the form of a raven, reminding me that my grandfather was with me or affirming what I was doing. Even when the day has been challenging, as soon as I am on the back of the sled with my dogs running down the trail, the stress of everyday life disappears. The stress is taken by the wind brushing across my forehead, and my whole being is focused on the dogs. I am in sync with the dogs. The team and I race toward nature’s escape.* The further we get from town, the lighter we feel.

While it was dark out, it was still light enough to distinguish the features along the black tree silhouettes against the navy-blue night sky. *The sound of my sled gliding across the compact snow echoes through the boreal forest and into the homeland of Dieł Taaneets (Rampart). I feel the historical and humbling energy of those that rode these trails before me.* As I follow behind Joe, I see what appears to be a mystifyingly large raven sitting atop a tree. Immediately, I think to myself, how odd to see a raven in this dark. *Just ahead of the dogs, spruce trees form a tall arch. In my mind’s eye I imagine that the arch is the finish line of my dream race*. The raven leaps from its perch to avoid the commotion below. As I watch the raven leap from atop the tree, it seemingly disappears into the black silhouette of the tree line. I wait, expecting to see the raven’s silhouette rise above the trees, but see nothing. *A profound feeling of peace engulfs me; it is as if all my worries and questions have been answered. The young dogs are energized and show me what they can do; the team runs in unison. I had positioned the dogs just right. The stress is lifted, and a flow of positive energy trickles up my spine. I inhale the fresh air and exhale in relief. The light, fresh air charges my soul.* I scan through my thoughts for every humanly possible explanation for what I am seeing, but none align with my lifetime of observations. As I follow in Joe’s tracks, underneath where the raven sat, an immediate lightness fills the dark environment, it is as though time stopped and the globe went silent. In my mind, the unexplainably large raven and subsequent lightness could only rightfully be explained to be Joe’s late grandpa, George, watching his grandson with energy so proud, I could feel it.

When we arrive home, Joe’s new energy and weightlessness fills the air. I tell him that his grandpa was with him at one point. I freeze. I can still see and feel that point in my mind’s eye, where we cruised under the tall arched trees. I now know my grandpa is proud of me, and I am where I am supposed to be. Here I had thought that it was only me who felt time stop; however, in fact, that experience was so powerful that Joe felt it too. 

This story illustrates a culturally honed engagement with nature, holistically entwined with places, people, animals, and community. Nature is a space (beyond place) where values and skills for life are accessed, developed, and cemented. It is a teacher, a refuge in coping with the stresses of modern life, a bridge between generations, and between past, present, and future. Joe and I, our awareness and attunement cultivated by culture, felt Joe’s grandpa there. George Attla Jr.’s journey echoes the struggles and disruptions so many Indigenous peoples have faced, and their paths to reconnection.

### 1.2. George Attla Jr.’s Legacy

To the world, George was a renowned dog musher known as the Huslia Hustler, a title dubbed for only the most elite Huslia dog men, like Bergman Sam, Cue Bifelt, Bobby Vent, Warner Vent, and the original Huslia Hustler, Jimmy Huntington. George’s mushing legacy is commemorated in books [1], the 1970s motion picture, Spirit of the Wind [2], the documentary ATTLA [3], and countless interviews and articles spanning the 1950s to the mid-2010s. George is the winningest dog musher in history and will likely remain so. Even so, less attention is drawn to the foreground of his mushing legacy.

While it is common knowledge that George’s knee was fused in childhood to combat tuberculosis, it is less known that he spent half his childhood in Sitka, Alaska, nearly 1000 miles away from his family, his culture, and the land that nurtured him. At age 8, George was diagnosed with tuberculosis and sent to Mt. Edgecumbe Hospital, alone, to receive treatment. It was mandated that he stay in Sitka to gain an education at the local boarding school, where he remained until he was 17. Excitement boiled inside him when he was finally permitted to return home; however, soon after, he quickly realized the stark reality of being severed from his culture. He had forgotten his first language, forgotten how to trap and fish, and been alienated from his birth right to coming of age hunting rituals and knowledge; his own siblings had to reintroduce themselves to him. He spent years in a disoriented state, lost between two worlds. However, despite the time and connections he lost, sled dogs remained the same.

Elders saw his internal struggle, similar to that of a war veteran emotionally shocked in coming home to face unexpected life changes. They gladly welcomed the opportunity to help him reconnect to culture as his reinterest in sled dogs bloomed. George considered the Elders his professors and eagerly sought their guidance and friendship. The dogs served as a bridge between generations, but also closed the cultural gap George was trapped in. The Elders taught traditional knowledge without judgment and George learned without stress. He naturally enjoyed paying attention to each experience with the dogs and also when on the land. He sought to understand more by pondering Elder’s stories in order to apply them to his life and fine-tune his skills. On his own terms, a stronger inner confidence and champion mindset was cast for life.

This paper and analysis serve not to prove what the people of Huslia and other dog mushing communities already know, but to help Indigenous peoples get back to the way it was meant to be. Indigenous communities were once filled with a thriving and vibrant society, firmly grounded in their relationships to all of creation. Stories speak to these relationships, helping to frame this article and provide a context for all that follows. The stories above, and the current article aim to highlight an enduring cultural conditioned astuteness, reverence, and reciprocity for nature. This cultural conditioning fuels vitality in Native communities by instilling purpose, a whole sense of self, ownership, strong will, self-confidence, and more. George Attla envisioned the Frank Attla Youth and Sled Dog Care—Mushing Program (FAYSDP) to bring back this vitality. He shared that his imagined FAYSDP was both tested and developed by the interest of Huslia’s youths and community in the moment he shared his vision with them. The resulting excitement showed him that traditional and time-tested cultural revitalization was possible still.

The FAYSDP sought to teach youths sled dog husbandry, veterinary science, cultural values, and practices under the guidance and mentorship of Elders, role models, and dog mushers in partnership with the school. Huslia’s legendary sprint sled dog racing champion and program founder, the late George Attla Jr., strongly believed that sled dogs and their rich cultural history were key to helping youths become and remain versatile and healthy-minded. In collaboration with the village of Huslia, and with help from his partner Kathy Turco, Attla developed FAYSDP in 2012 to try to remedy the social disruption felt by his people, that he knew all too well. The foundation of the program is youths’ hands-on work with sled dogs. A yard of sled dogs offers young and old a positive and enriching emotional experience without fear of human judgment. The immediate acceptance that a happy and healthy sled dog offers helps youths feel good about themselves. The program design encourages the development of pride by learning from dedicated and experienced mentors, dogmen, and Elders. Kennel owners, the school staff, and community volunteers work together to plan the dog kennel field classes. Students learn about dog care including feeding, cleaning a dog yard, and monitoring overall dog health. Students also develop dog handling and mushing skills including harnessing and hooking up dogs, training a team, and racing.

The goal of this project is to understand the depth of youth and community perspectives on the FAYSDP’s impact. Due to the program’s holistic nature, it is important to understand and appreciate the impacts of the program in an Indigenous lens. Thus, we review Indigenous worldviews that provide valuable context for these goals.

### 1.3. Indigenous Worldviews

Taking a line from Anishinabe scholar, Joan McGregor, “one disclaimer must be made about Indigenous views of the natural world: there is no monolithic Indigenous belief system dictating correct treatment of nature. Nevertheless, some general similarities in worldviews, conceptions, values, and epistemic approaches in Native views about their relationships to the Earth exist, and they can provide fruitful ways of thinking about humankind and nature” [4] (p. 111). The following is written from a Koyukon Athabascan perspective to illuminate Koyukon Athabascan knowledge, and the understandings from other Indigenous cultures are integrated throughout because they contextualize a Koyukon worldview: We recognize that “no short answer exists” for defining Indigenous knowledge due to the diversity and complexity across Indigenous communities [5] (p. 35).

Athabascan people inhabit Interior Alaska and into Western Canada. Their interdependence with nature was born in the Creation Story. According to *Kk’adonts’idnee* (the Koyukon Athabascan creation story), Raven created everything on Earth, and initially, as humans who were able to freely transform between human and a singular nonhuman form. Animals, plants, rivers, trees, even rocks and weather phenomena, etc. are all considered Raven’s descendants and are, thus, interrelated and interconnected. As Raven’s descendants, they each have a spirit and are revered as having inherent agency, consciousness, and sovereignty. According to *Kk’adonts’idnee*, when Raven first created everything on Earth, Raven felt life was too easy for humans. Thus, comically, Raven created life as we know it today, fixing some in their nonhuman form. As a result, humans had to watch and learn how plants and animals interacted with their landscape and reacted to weather patterns.

While Indigenous creation stories vary culture to culture, they maintain similar concepts. Creator’s descendants themselves guided the first Indigenous peoples to study and learn the intimate interconnections between them through their lives and interactions. These early Indigenous cultures could not avoid these relationships as they were constantly associated with and depended on the land for food, shelter, clothing, medicine, tools, etc. Over many generations, human beings recognized that selfless collaboration with the environment and each other manifested fruitful outcomes for human and nonhuman alike. Indigenous peoples evolved with this organic and holistic way of seeing and relating to the world, themselves, and others (human and nonhuman). Tewa Pueblo scholar, Gregory Cajete, believes that all Indigenous peoples share this basic premise on interconnection, and “through the seeking, making, sharing, and celebrating of these natural relationships, they came to perceive themselves as living in a sea of relationships” [6] (p. 178). Therefore, Indigenous peoples consider words like “nature” and “environment” to encompass humans and their kin-like relationships to all that “nature” is.

To honor these relationships, Indigenous peoples philosophically developed their own spirituality, recognizing reciprocal interrelations [7]. To pay gratitude (a deliberate input) for the environments’ generosity, “Native peoples developed many rituals and ceremonies with respect to motherhood and child rearing, care of animals, hunting and trapping practices, and related ceremonies for maintaining balance between the human, natural, and spiritual realms” [8] (pp. 8–9). Yup’ik scholar Angayuqaq Oscar Kawagley considered all life to be renewable and entirely self-sustaining should the relationships be cared for according to traditional practices (specifically the knowledge therein) that evolved over thousands of years. Clear-cut examples of some of these practices include not taking more resources than were necessary, such as not gathering all the berries from an entire branch or area, or traditional governance against extractive practices. Yet, these practices also include an essence of respecting an animal’s consciousness and sovereign decision to “give itself” in sacrifice to you and your family as a means to pay homage in reciprocity.

To maintain these relationships, Indigenous peoples’ life endeavor was to keep harmony within these relationships; “the real test of living was to be able to establish a harmonious relationship with that perfect nature—to understand it, to see it as a source of one’s life and livelihood, and the source of one’s essential spiritual being” [6] (p. 179). To do so, one must recognize that “respect and regard are essential to strong relationships” [7] (p. 90); therefore, “one must acknowledge and take pride in a relationship” [8] (p. 11). In essence, maintaining these relationships is the foundation of Indigenous knowledge. Thus, one can only achieve attunement by holding reverence for all creation, which is kin, embedded with consciousness and agency, as well as through astute awareness of the creative consciousness within creation and under exclusively reciprocal terms entrusted by Creator.

Essentially, to produce knowledge under the Indigenous knowledge system, you must revere all creation for its conscious energy (or spirit) within and be ever aware of their interactions in the same way you would your own bloodline. You must acknowledge that Creator bestowed this responsibility onto you and all of the Creator’s descendants. You must either acknowledge these terms or your worldview passively asserts and abides by these terms. Indigenous knowledge and its production are shaped by an Indigenous worldview, just as Western knowledge and research are shaped by its worldview, according to their ontologies, epistemology, axiology, and methodology [9]. An Indigenous ontology and epistemology are focused on relationships (human and nonhuman), whereas Western ontologies are focused on individual separated objects. Opaskwayak Cree scholar, Shawn Wilson, illustrates this point by tracing the difference in Cree and English language orientations; in the Cree language, pen literally translates to “something that you write with”. To emphasize this point, he states that, “objects themselves are not named; rather, what they might be used for is described” [9] (p. 73). An Indigenous axiology is being accountable to these relationships, living up to one’s role or obligation. This is in part parallel to the methodology, which is guided by “the vested interest” of respect and reciprocity, or the “usefulness of the result(s)” to the community [9] (p. 77).

Derived from their holistic worldview, Indigenous peoples consider an empirical “result” to be only one facet of their knowledge, proclaiming that Indigenous knowledge is not simply descriptive. Indigenous knowledges (plural) are interwoven with the people and places they are produced within. Therefore, there are many empirical products produced by and belonging to many specific Indigenous groups. Indigenous knowledge as a paradigm includes how Indigenous peoples come to know through stories, lived experiences, and their own personal development and understanding as their comprehension advances; thus, the Indigenous knowledge paradigm applies generally across all Indigenous peoples because they hold a similar worldview and epistemic approaches [10].

Indigenous knowledges are place-specific knowledge gained by experience, but also a source of moral knowledge that provides guidance and awareness for how to treat and sustain relationships with nature [4]. Indigenous knowledge seeks to better understand dynamic relationships in nature in an attempt to keep harmony and balance in the “sea of relationships”. Importantly, Indigenous knowledge asserts that knowledge is alive and adaptive, “that the world is in motion, that things are constantly undergoing processes of transformation, deformation, and restoration, and that essence of life and being is movement” [11] (p. 48). In recognizing that all nonhuman life possesses inherent agency granted by Creator, humans are entrusted with “tuning in” to and hyper-focusing on the subtleties of nature to be conscious of potentially significant consequences. This “tuning in” evolves and calibrates along a lifetime’s worth of observation, modeling role models’ behaviors and few explicit instructions.

Blackfoot researcher and educator, Leroy Little Bear, maintains that, across Indigenous knowledge, “the most important aspect of traditional knowledge is its philosophical and/or paradigmatic base” [12] (p. 521). He argues that all sense datum is interpreted through philosophy and paradigm to form Indigenous reality. In parallel, Cajete affirms that the body is attuned to subtle forces of nature through smell, sight, taste, touch, and sound, and that Indigenous peoples are adept at “thinking” with their senses. Indigenous peoples’ sensual experience with nature is energized by a time-honored and cultural conditioned “tuning in” that heightens their sensitivity and awareness to subtle qualities and breadth of nature [6] (p. 20). Cajete argued that this sensual experience is not “supernatural” or “extraordinary”, but that all people are born with this basic acuity. Western societies abstract the body from the mind with their overt focus on objectifying and rationalizing experience, thus eliminating the intimate experience with nature. Training our bodies to absorb the subtle energy within nature and enabling our minds to capture it as thought is a lifelong study. Information absorbed through the body, in tandem with knowledge from lived experience and story, are syphoned together and processed through moral obligations to maintain balance. Through this process of recognizing inherent agency and consciousness, Indigenous peoples cultivated a way of learning from the vast array of relationships around them. This holistic worldview based in kinship shapes Indigenous values, spirituality, proper behavior and ethics, ways of relating, and the basis of teaching and learning. Traditional values, including respect for land and nature, connection to animals, and spirituality are taught though subsistence practices [13]. Indigenous traditions of teaching and learning, centered in community, contribute to adaptability.

### 1.4. Indigenous Place-Based Learning and Wellbeing

Traditional education processes were carefully cultivated around observing natural processes, constant inquisition, and adapting understandings [14]. Western education processes emphasize written knowledge used by dominant societies to further their goals and facilitate upward economic and societal status. However, Indigenous teaching has traditionally taken place through oral, visual, and experiential means that are driven by the Indigenous cultural values and place [15]. Traditional Indigenous education involves extended family, implicitly emphasizing application and imitation, as well as sharing and cooperation [16]. Additionally, Indigenous peoples recognized life as constantly in flux, where no one occurrence is ever 100% repeatable; through countless generations, they accumulated general patterns and predictions for these observable interactions [17]. In short, they never stopped learning because life around them was always changing. They fostered an atmosphere of constant reflection and reconsideration, where everyone was constantly learning. Fourteen traditional teaching practices that commonly occur within most Alaska Native cultures include Earth-based pace, attending to relationship, placed-based knowledge/learning from the earth, learning/thinking/working as a group, learning from Elders, close observation and emulation, indirect teaching, silence, pausing and reflection, all senses experiential learning, visual/nonverbal learning, storytelling, dances and games, good instructions, and humor [18]. In traditional cultures, the greatest learning comes from place as it is important to know; it means adding your knowledge to that which is passed on to you from the Elders. Additionally, the promotion of traditional knowledge has been shown to increase social, cultural, and symbolic capital [19].

Unfortunately, Indigenous communities worldwide live within a long legacy of environmental dispossession, through direct, physical separation from the land and indirect implementation of policy that destabilize relationships Indigenous peoples have with the land [20]. Richman and Ross refer to environmental dispossession as “the processes through which Aboriginal people’s access to the resources of their traditional environments is reduced” [21] (p. 403). Land claims, forced assimilation into fixed communities, and the cumulative effects of climate change have eroded Alaska Native people’s gateway to the land. Alaska Native peoples also shoulder the indirect dispossession of land through the dark history of child abduction and forced boarding schools, assimilation into cash economies, and state regulation and restriction of Alaska Native traditional harvesting practices [22]. Direct and indirect forms of land dispossession have resulted in their own myriad of individual and community health detriments, all of which sit atop of access to equipment and capital for subsistence activities. Direct relationship with nature is the only way to maintain this knowledge and build upon it. Self-determination is important for wellness in Indigenous communities and is linked to protection from suicide [23].

Indigenous people in the Yukon Flats described important elements of individual and community wellbeing [24]. Elements of individual wellbeing include achieving goals, staying busy, spirituality, passing on culture and traditions, and developing a strong identity as a Native person. Major elements of community wellbeing include taking care of one another, working together, and self-sufficiency. Similar elements of wellness have emerged through discussion groups and interviews in other Alaska Native populations [25,26,27], with strong interconnections between these themes. For example, in one study, family was interconnected with traditional knowledge and values, and subsistence, which were closely linked with access to resources [25]. Connection to family was a central coping strategy that was also linked with opening one’s frame of mind [26]. Individual acts of self, such as setting goals, seeking education, and working toward positive change, was intertwined with providing for one’s family and community, as well as Indigenous values such as hard work and sharing [25]. Individual and relational wellness was rooted in cultural and spiritual values [27]. Yet, both self-determination and the traditional practices so intertwined with Indigenous values and spirituality are threatened.

Interventions that resonate with these Indigenous ways of learning and being are likely to be more effective. It is important for such interventions to build from local Indigenous strengths, systems, and settings [28]. Place-based interventions that integrate experiential embodied learning can facilitate deeper reflections, insights, cultural connections, and health behavior change in AI/AN communities [29]. In the Western world, “place” is often boiled down to a dot on map, longitude and latitude coordinates, and/or colonized names. For many Native peoples, you would likely struggle to find a common map that denotes our traditional settlements. For Indigenous peoples, “place” is subjective; our sense of place is grounded in the nature that surrounds us. Furthermore, kinship worldviews instill a moral commitment to care for all of creation. To even entertain this obligation, we must actively participate with nature and strive to understand these intricate interrelationships among all of creation. Sense of place, identity, and belonging are grounded in these relationships and patterns studied over countless generations.

### 1.5. Digital Storytelling and Cultural Connection

Technology and digital storytelling can be utilized to assist in sharing Indigenous knowledge. Digital storytelling resonates with oral narrative traditions and integrates modern technology in culturally resonant ways. Digital stories can build intergenerational connections, and communicate and celebrate cultural knowledge, values, and identities [30,31,32,33]. They can also facilitate a deeper understanding of participants’ lives, needs, challenges, and strengths, as well as the effects of programs or interventions [33,34,35]. Digital stories have been used to communicate local lived experiences on the interrelationships of land and water with individual and community health [36] and express Indigenous youths’ personal connections to place and environmental challenges [37]. Other visual methodologies, including photovoice, have also illustrated Indigenous youths’ connections to nature, and the ways nature serves as a source of wellbeing, cultural identity, purpose, and a relational conduit to others human and nonhuman [38].

The connections that digital stories offer are important, as connection to culture is protective, particularly in settings of cultural disruption [26,39]. Opportunities to live out cultural values, express reciprocity, nurture relationships, carry out traditional harvesting practices, and contribute to family and community facilitate competence and resilience for Alaska Native youths in the face of challenges [40]. Cultural identity conflicts in Alaska Native youths have been identified as an impediment to the vision of a healthy future and are sometimes buried beneath the raw forms of alcoholism, suicide, or violence [41]. However, cultural connection can heal such ruptures and build resilience and a sense of purpose, highlighting the importance of promoting cultural identity for Indigenous youths [42]. Fostering traditional values, cultural identity, and spirituality increases wellbeing in Indigenous communities [13,43], emphasizing the practicality of culturally based programs that connect youths to their place, ways of being, and culture.

### 1.6. The Current Study

Digital stories produced by students participating in the school-based community-driven FAYSDP offered an extraordinary opportunity to share a significant understanding of Koyukon Athabascan culture. Through a community-based participatory research partnership with Huslia and the Jimmy Huntington School, we examine how the FAYSDP affects youths and how connections to people, the landscape, dogs, and culture promote wellbeing. We ask the following research questions: How does culturally appropriate active engagement foster sense of place and promote perseverance skills? How does the cultivation of a sense of place and perseverance skills promote wellbeing? We address these questions by examining Huslia youths’ and community perspectives on FAYSDP, through photos and digital stories that youths created and focus groups with adults in the community.

## 2. Materials and Methods

### 2.1. Partnership

This project was a collaboration between school and tribal partners in the community of Huslia and investigators and students at the University of Alaska Fairbanks Center for Alaska Native Health Research. The collaboration first developed between the research principal investigator (Jacques Philip) and tribal members over the course of prior projects, and then continued to expand, with investigators from psychology and rural development disciplines, and Athabascan university students joining the team. The project would not have taken place without the community wanting it to happen. The principal investigator sought recommendations and guidance from the Huslia Tribal Council and other community members regarding who would be important to include in the community planning group (CPG). The CPG that mobilized to direct the research comprised four members—one from each of the following: Huslia Tribal Council, Jimmy Huntington School, active in the FAYSDP, and an individual from the community’s wellness team. The CPG was established to guide the research and ensure cultural appropriateness for all steps of the project. Teachers also provided guidance for integration of the research activities into their classes. See [19] for additional information on the partnership and the project. The research was approved by the University of Alaska Fairbanks Institutional Review Board, as well as the CPG, Huslia Tribal Council, and Jimmy Huntington School in Huslia. The research team was in contact with the CPG throughout the research project and publications and continues to meet with them to further discuss interpretations of the project findings and plan additional research addressing their priorities.

### 2.2. Setting

Huslia is on the Koyukuk River in the Western Interior of Alaska. It is one of few growing Athabascan remote villages. The 2020 census showed that Huslia had 275 people [44], and 91 are enrolled in the local Jimmy Huntington School [45]. There are no roads connecting Huslia to other communities; hence, travel to and from the village must be achieved by plane, boat, snow machine, or dog team. In addition, traveling and shipping goods to Huslia are costly, making any travel and supplying limited. For example, in 2022, one round trip airline ticket to Fairbanks (the closest city) costed 400 USD, rural gasoline was upward of 7 USD/gallon, and the price of freight from Fairbanks was 1 USD/pound, meaning that a 40 lb. bag of commercial dog food was a minimum of 100 USD including 40 USD for air freight.

The community is grounded in dog mushing and surrounded by about 50 miles of established and maintained mushing trails and countless miles of additional trail possibilities. An Alaska Department of Fish and Game, Division of Subsistence report [46] conveyed that the viability of rural sled dog teams was dependent on local resources. For Interior Alaska Athabascan families, sled dogs have been an integral part of survival for as long as anyone can remember. Today, dogs continue to have a purpose and importance, and their year-round care takes the committed work of an entire family.

### 2.3. Participants

In the fall of 2017, the research team invited all middle- and high-school students to participate in photovoice and digital storytelling workshops. All 10 middle-school students, ages ranging 12–13, agreed to participate in the photovoice and digital story workshop. Five of about 10 high-school students, ages 14–16, were interested in the digital story workshop. Fourteen of the 15 participants identified as Athabascan, and one identified as Inupiaq. Six participants identified as female, while the remaining nine identified as males. The research team collected youth assent and parental consent from each of the participants.

### 2.4. Procedures

#### 2.4.1. Youth Photovoice and Digital Storytelling Sessions

Photovoice and digital storytelling workshops are qualitative methodologies following principles established by Community Based Participatory Research [47]. This type of qualitative research helps and encourages participants to reflect on, create, and share their stories, with the objective of being heard by policymakers [34,47,48]. The photovoice and digital storytelling sessions were integrated into middle- and high-school classes, with some activities continuing after school. Teachers prepared the students for the workshops, helped facilitate the process, and connected the activities to their curriculum and lesson plans.

**Figure 1:**
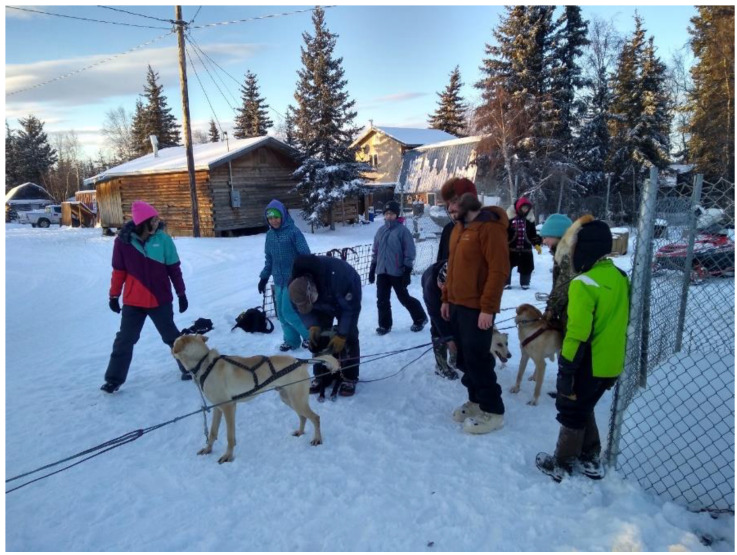
“It’s a Good Class. Class harnessing team. Shows everyone helping. I like seeing everyone”.

**Figure 2:**
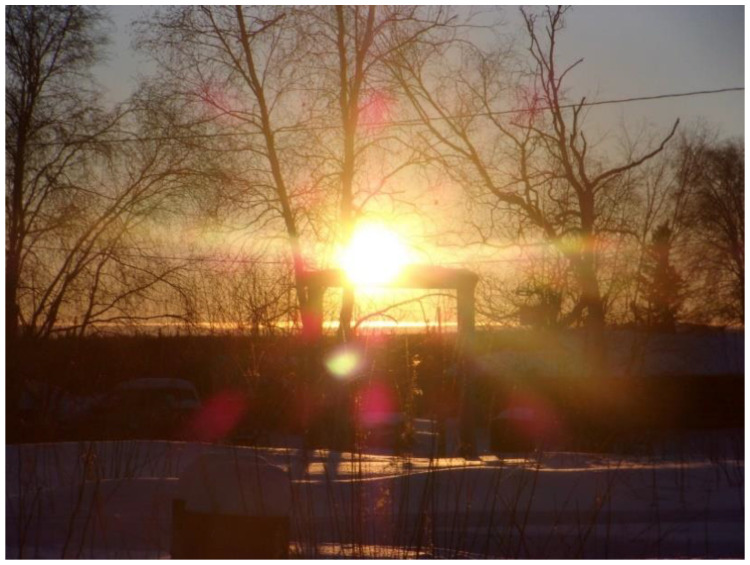
“Hello Sunset. The colorful sunset. It looks beautiful and colorful. The fish rack is holding the sun”.

To begin photovoice sessions, the research team conducted training on ethical photography, technical review of camera handling, taking photos, and storage. The research team tasked the middle-school students with taking photographs they felt were representative of what FAYSDP meant to them and how it affected their community (see Figure 1 and Figure 2). The youths used different sources for the photos (including cameras provided by the research team, their own phones or devices, and photos taken by others with permission to share).

The youths described what their photos meant to them and organized the collection of photos into themes (see Appendix A for an example). Photovoice workshops later included discussion sessions following guidelines established by the ORID (objective, reflective, interpretive, decisional) framework [49]. This process was locally adapted by the Athabascan university students, who linked youth reflections (on what they see in the collection of photo themes, what they feel, insights and inspirations, and how they can use and share the learning) to four metaphorical connections to culturally relevant communities and land: east (Hughes), south (Koyukuk), west (Nome), and north (Hot Springs).

The team then facilitated digital storytelling workshops with both the middle- and the high-school groups. Digital storytelling uses voiceover, images, and music to share a personal story [34]. These workshops use digital stories to build a narrative that reflects the participants’ views of the FAYSDP. Facilitators encouraged the youths to brainstorm the story they wanted to convey based on their perception of the FAYSDP, and then share their thoughts with their peers to further refine their message in story-circle discussions. Following story-circle discussions, the youths each created their own scripts and storyboards, which served as a visual method to line up words with photos. Some youths chose photos from the photographs taken for photovoice sessions, along with other personal photos the participants felt were representative of their story. After recording voice-over and uploading photos to WeVideo [50], participants toyed with their storyboards and music to match until they were satisfied with their story. Wevideo is an online platform that offers music, transitions, and editing capabilities ideal for our study. After the youths were finished with their stories, all participants and their parents consented to share them. Facilitators invited the community to attend a showing of the 15 digital stories at the community hall with the participation of 53 Huslia members. The youths’ photovoice and digital stories can be viewed on the ACHILL and CANHR websites [51,52,53].

#### 2.4.2. Adult Focus Groups

The team later disseminated the findings of youths’ digital stories to the CPG members and in a broader community celebratory gathering in Huslia. The team and CPG then engaged community members to take part in focus groups to further discuss findings. Nineteen participants (11 women and eight men; 18 Alaska Native) took part in two focus groups. Community members shared their thoughts on the youths’ stories and research findings, how what they saw fit with their experiences and observations, and other ways they felt the program affected youths, the school, the community as a whole, relationships within and across generations and communities, and sense of connection to Huslia. They discussed the main challenges of the program, and what they saw as the most important effects of the program. This process of triangulation helped to both validate and contextualize the findings on youths’ perspectives of the program, integrating viewpoints from adults who have observed changes in the community over time, and facilitating a broader perspective on findings.

### 2.5. Analyses

Qualitative analysis and data management was performed using ATLAS.ti [54] on the youths’ photovoice and digital stories and adult focus groups. Qualitative analysis aims to interpret the meaning and context of data. The research team used both deductive and inductive approaches to analyze the data (digital stories and scripts). To develop the codebook, we used Athabascan Cultural Values established by the Denakkanaaga Elders Conference [55] and deductive codes comprising themes from existing theoretical models of social capital provided by Schaefer-McDaniel’s framework [56]. Themes representing connection to place were also included in both the cultural values (e.g., respect for land and nature) and social capital codes (e.g., sense of belonging/place attachments). During photovoice sessions, the participants helped develop inductive codes that they saw as emerging themes in the photos. These were also added to the codebook. Lastly, the research team also identified inductive codes derived from emerging themes from the digital story scripts. See our previous publication [19] for the codebook.

After the codebook had been developed, defined, refined, and finalized, the team coded the scripts independently, and discussed their individual findings later as a group. Any code discrepancies were discussed to consensus by the team. Trustworthiness of data was established using multiple perspectives in analysis and multiple coders [57]. Furthermore, the team comprised three faculty members who had experience in dog mushing, social capital, qualitative analysis, digital stories, and photovoice, and two Koyukon Athabascan student researchers who had experience in Athabascan culture and values.

## 3. Results

As noted previously, Indigenous knowledges are more than factual knowledge; they are dynamic, cyclical, and interwoven, as they are embedded in the places and people (and their development), and include traditional education processes and empirical knowledge [10]. Therefore, the below-described broad spheres do not have solid, well-defined borders; instead, many themes overlap or can be labeled to different spheres depending on the reader’s perspective and experience. For example, the Athabascan Cultural Value Practice of Native Traditions cannot be restricted to one system. With that in mind, this analysis does not serve to delineate and define Indigenous knowledge spheres. Three overarching and generalizable interrelated knowledge spheres are present in the FAYSDP: connections to dogs, the environment, and people.

**Figure 3:**
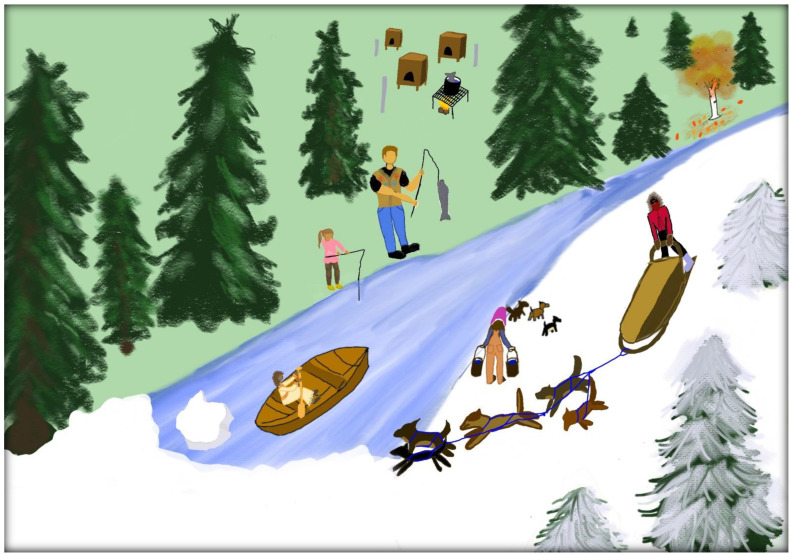
Relationship spheres: Connections to dogs, the environment, and people (Laura Ekada).

The image in Figure 3 illustrates these spheres. The drawing was created by Laura Ekada with the help and guidance from Hailee Tanner. The image is an example of the Indigenous worldview highlighting the interconnections between people (intergenerational and community), the environment (intimate understandings of seasons changing), and animals from a bird’s eye perspective. The health of the environment, animals, and humans all depend on each other. Caring for sled dogs is hard work. In the drawing, there is a man and a young girl fishing so that they can have food for the dog team. The two people fishing are a representation of the intergenerational relationships that are strengthened through caring for sled dogs. In the background of the drawing, there is a dog yard with a fish in the soup pot for dogs to eat. On the other side of the river, there is a woman hauling water for dogs. In the river there is a woman canoeing in the river to represent the importance of spending time in the environment. Living in a village on the river means that you have to be skilled at traveling the river and know how to get where you are going. In the image, the four seasons are represented. The seasons are not evenly distributed because, in Alaska, spring and fall are very short seasons. The right bottom corner of the drawing is wintertime and dog mushing season. The left bottom corner is springtime and can be noted by the ice breaking up. The top left corner is summertime when the fish run in the river. The top right corner is fall time when the birch trees change colors. The clothes of the people in the drawing represent their Indigenous heritage. The man is wearing a beaded vest that is lined with beaver fur. The little girl fishing is wearing a bets’egh hoolahn (Koyukon Athabascan word for kuspuk). The woman canoeing is wearing a deer hide dress with a beaded moose hide belt and beaded moose hide collar. The person dog mushing is wearing a red parka with a wolf and wolverine ruff and beaver mittens. The woman hauling water is wearing a rabbit fur hat.

### 3.1. Dog Relationships

In their photos and digital stories, youths shared what dogs meant to them and their community. They expressed how their personal, family, and community connections to sled dogs fostered pride, joy, peace, relationships, and important values and lessons for life. Adult community members echoed and expanded on the ways connecting with and caring for sled dogs affect youths into the future.

#### 3.1.1. Cultural Upbringing Sparks Interest in Sled Dogs

The youths expressed an innate interest in sled dogs. They talked about how they helped their grandparent or a local dog musher with their dogs, watched community dog races, liked learning new things about sled dogs, and expressed pride in a family member winning or placing in a prominent race. Youths often expressed that participating or watching these activities is what sparked their interest in sled dogs and the FAYSDP. Two middle-school boys reflect on their grandfathers’ achievements:

*My grandpa Warner Vent was an Iditarod musher; in the early 1970s, he won second place twice. He is the one who has really inspired me to get involved with dogs and this program.*

*So, my late grandpa Cue Bifelt won the Open North American Championship sled dog race, which is one of the biggest sprint dog races in the world. The Frank Attla Program taught me how to work with dogs and learned some history from where I come from.*

#### 3.1.2. Youths View Sled Dogs as Welcoming and Capable of Educating

Youths often discussed the joy and happiness they felt when engaging with sled dogs and puppies. Youths recognized that sled dogs have their own personhood, making it easier to empathize and relate to them. Through this, they learn how to consider things through a different perspective than their own. A middle-school boy wrote: *What I like about dogs is that they all have their own personalities. Dogs make people feel happy.*

Youths described sled dogs and puppies as *trustworthy, adorable, respectful* and as supportive. One middle-school boy wrote: *Dogs are always there when you don’t have anyone to talk to you.*

Adults also saw that sled dogs made youths feel comfortable and that actively engaging with them gave the youths a sense of purpose and self-esteem. One community member reflected on her two sons’ experience in the FAYSDP and shared the following:

*Personally, both my boys have been in the program, and my boys are really quiet, and they’re really shy. And T, I was even shocked that T did a video. He’s never done anything like that before. And so, it does make a huge difference on their self-esteem. H, when he was in high school, he was shy, and he wouldn’t open up to nobody. But when he went in that dog yard, he was all aboard, you know. And it came, it brought him out of his shell. So, it does make a big difference all the way around.*

Youths often discussed the joy that dogs bring them while simultaneously discussing the skills sled dogs themselves taught youths. The youths view sled dogs as capable of teaching life lessons, which insinuates the youths are receptive to learning from different perspectives, adept at learning through observing, and cognizant of changes. One middle-school girl wrote: *Dogs have educated me with obedience, love, loyalty; they gave me company, taught me how to get right back up, they made me tougher and smarter.*

Through working with and running sled dogs, youths learn to take on challenges, get outside their “comfort zone,” and set goals. Setting goals implies orienting your mind around a future objective and consciously analyzing behavior that detracts or promotes said goals. A high-school boy wrote: *Dogs teach you that you always have to work hard. The key is to never give up and that has also been very true to me. You can achieve anything when you can put your shoulders into it.*

Another youth shared: *Dogs will always try their best in everything they do. They never give up or will slow down. It’s almost like they don’t know how to, and they always know how to calm and relax whoever is with them.*

As illustrated here, in addition to teaching youths how to work hard, sled dogs taught them how to remain calm in stressful situations, such as running sled dogs and competing in races. Running a team of sled dogs requires the youth musher to monitor their surroundings and obstacles, monitor each of the dog’s body language, and ensure the towline is taut to avoid injuring the dogs. In competition, the youth mushers must monitor the same three things, all while being aware of their competitors (and their dogs), and actually competing in the race itself. In what begins as mayhem, youths often quickly learn to manage all the moving parts and persevere by being in the landscape and watching how the dogs calmly contain their excitement. A middle school girl wrote:

*I finally raced in a four-dog race at the carnival a couple years ago. I was very excited I remember thinking that I was certainly going to fall over or tip. But it turns out I didn’t, and running with the dogs was very peaceful.*

#### 3.1.3. Lessons in Caring for Sled Dogs and in Kennel Maintenance

The youths acknowledged that caring for sled dogs taught them how to work hard and never give up. The following quote by a middle-school boy is a well-rounded explanation of the amount of work necessary to care for sled dogs:

*There is always work that needs to be done, cleaning poop around the dogs and puppies, chopping wood for the dog pot, cooking the dog pot, feeding and watering the dogs, and a whole lot more to do. I’m one of the few lucky kids that has a parent or grandparent that has a dog yard. It’s a lot of work, but that is also a good thing, it helps to teach you what hard work is, and also helps to have a better work ethic. It definitely fills the day, taking wet, peed on grass out of houses and putting fresh, dry grass in their houses so they can stay warm and healthy. Running them, cutting their nails, checking their arms and legs to see if they’re hurt or sore. Dry off harnesses and gang lines, making sure you got everything, hooks, harnesses, ganglines and necklines. You also have to run the dogs daily, keep them healthy and in shape. Which also reminds me to stay healthy and in shape myself. If dogs don’t run, they start getting depressed, and start getting sick, and start losing weight.*

As evident here, youths often associated the many tasks and responsibilities with positive feelings toward their future and appreciation. Youths also asserted that, in the dog yards, they learned teamwork and gained a sense of unity by “creating bonds” among their peers. The following are several youths’ reflections for dog yard photos from the photovoice activity:

*Family, culture, pride. I see our history, our elders and teachers. I see family and friends, and teamwork.*

*Getting ready to feed dogs. All working together. Happy people.*

*I feel happy that dog yards still make people happy.*

*We are socializing with the puppies, having fun. Learning how to coach puppies. I love that we’re working together to help these puppies and teach them how to race and listen good.*

#### 3.1.4. Sled Dogs Spark Intergenerational Relationships

Sled dogs are a culturally significant common ground that adults and Elders can utilize to establish relationships with youths, and vice versa. Because youths were naturally attracted to sled dogs, and their inviting demeanor was uplifting, youths seemed to feel more comfortable initiating interpersonal relationships. A high-school boy shared:

*Kathy Turco and George Attla always liked working with the community… They always liked to help with my little brothers after my dad gave away his dogs. They always let my brother come over to visit their dogs, even when my brothers missed their puppies. I remember Kathy letting us play with their puppies. They even let my brothers feed their dogs and I always saw them come home with big smiles saying that they had fed their dogs.*

Youths also shared how the FAYSDP deepened relationships. This is elaborated on in more depth in Section 3. A middle school boy shared: *I’m thankful for this program helping me get closer to my grandpa and his dogs that I raced and helped with.*

### 3.2. Environment

The youths expressed their attachment to their space and place, and all that was nested within it, including their identity, community, culture, land, and nature. Dog care and mushing fostered a sense of community identity, helped youths experience the landscapes surrounding their home, facilitated an understanding of different perspectives and ways of doing things, brought the community together, and connected youths to their culture, community, and self-envisioned future.

#### 3.2.1. Huslia Attachment and How the Youths Define Huslia

Almost all the youths identified themselves as being from Huslia. They recognized nature and dog mushing as part of their personal and community identity. Two middle-school boys introduced themselves and their connection to Huslia, describing it as follows:

*Hi, my name is Jordan Vent welcome to a place that is surrounded by forests and rivers, called Huslia Alaska.*

*Hi, my name is Jeremiah Henry. I’m from Huslia, Alaska, a place that is rich in dog mushing.*

A middle-school girl also highlighted the long-term connection between Huslia and dog mushing: *I can’t imagine our community without dog mushing. Dog mushing has been here for generations; it’s a part of our village and culture, a huge part.*

The fact that youths characterize Huslia as they do indicates that youths identify sled dogs, dog mushing, the landscape, and the people as a part of the social fabric of their community, and they are, thus, equally relevant in constructing their sense of place. The following quote by a high-school girl illustrates how intimately intertwined these pieces are by distinguishing how she is of her physical, social, and cultural environment:

*JesCynthia David se’ooze, dehoon Denaakk’e helde Hedo’ketlno seeznee. Ts’aateydenaadekk’ohn Dehn hut’aanh eslaanh. My name is JesCynthia David while in Denaakk’e they call me Hedo’ketlno, and I am of the Huslia people.*

Language, culture, community, and place are interwoven into identity, and expressed in the ways the youths introduce themselves. This introduction, combining both Denaakk’e (Koyukon language) and English, spotlights the emphasis that Indigenous languages put on action and connection.

#### 3.2.2. Connection to Land

As noted, the elaborate trail system in Huslia provides the community with many miles of dog training trails interwoven within the community, nearby lakes, ebb and flows through frozen sloughs, black spruce, tundra, and low hills. The youths discussed how running sled dogs brings them into the land, where they can experience, establish, and expand upon a base understanding of their landscape. In addition, being on the land gives youths the opportunity to practice critical thinking skills by creating their own observations of nonhuman interrelationships using their five senses and to further develop their culturally conditioned intuition in nature. For example, a middle-school girl shared:

*When I go racing I always think I’m gonna tip or crash into a tree until I realize I can really trust the dogs. Mushing also brings me into nature and I watch the birds and look at the trees and other stuff.*

Adult focus group participants highlighted that running sled dogs within their homelands seemed to lift the youths’ spirits. A community member shared: *That one girl that she graduated, we let her drive our dogs. She said she never felt so good just being in that trail, and they are so calm, and just watching everything, watching the dogs, and how they run.*

#### 3.2.3. Things Learned at Different Dog Yards

The youths explained that each of the dog yards they visit is different from one another. In doing so they are acknowledging that the dog yards are different in likely several ways; layout, types of materials and foods used, ways of doing tasks like watering the dogs or getting the dogs ready to run, musher philosophy, and more. Overall, they are being exposed to diversity and learning perspective by accepting there are multiple ways of doing things. By accepting that there are many ways to achieve the same objective, youths learn adaptability and flexibility and expand their knowledge.

*We go to three different dog yards. The dog yards we go to are Wesley, Wilson, and Floyd’s dog yards. Each dog yard is different from the other. At Wesley’s dog yard we go running with the dogs. At Wilson’s dog yard, he tells us stories, and then we go outside and clean up the dogs; sometimes we run the puppies. At Floyd’s dog yard, we play with the puppies and cut food for the dogs. We sometimes harness the dogs and let Floyd run them while we wait and play with the puppies.*

In addition to sled dogs teaching youths about learning from different perspectives, youths can apply that skill to humans and recognize that even adults gain knowledge from different perspectives. Youths recognize that other people may have more effective ways of doing things and they can implement them into their own lives to be more successful. A high-school boy shared: *My dad is a dog musher and he has gotten a lot better since a couple years ago. He has learned a lot of things from other mushers, that is a lot of the reason right there.*

Adults reaffirmed this way of learning and highlighted that dogs were the common denominator between mentor and learner. A focus group participant shared:

*I’ve learned everything from talking to older people when I was younger how to race dogs. I’ve called on many people, asked them questions. George, there are other people, you know, they’re Cue Bifelt, Lester Erhart, Freddy Jordan, all the people I have known throughout the years. And I’ve learned from other people how to race dogs. Not saying I was the best dog musher in the world, but it made me better. I was able to communicate with older people because we had a common interest.*

Two of the dog mushers mentioned in this quote belong to another Koyukon Athabascan village, highlighting that sled dogs bridge relationships and mentorships through communities.

#### 3.2.4. Sled Dogs Bring Communities Together

The youths discussed how sled dogs and community dog races bring together Elders, youths, locals, and even other communities. For example, a youth noted how the FAYSDP *“helps us be community. We get to look at how it all started. We get to work together.”* A youth shared: *So, it makes the town more alive. So, when dog races happen it bring people together from other villages, or just the village.*

Sled dogs are a culturally relevant way to learn and get youths engaged. Student interest also stimulates community involvement and cohesion. In the focus groups, adults expanded on the ways that youth engagement spirals into widespread community connections:

*Students that go through the program, they start learning about dogs, and it sparks the interest for them, the children. So, when the children have an interest in something, you know, the parents tend to want to support their interest or what not… But when you start seeing your children have an interest in it, you tend, as a community member, the community, to get more involved.*

#### 3.2.5. A-CHILL Good for Huslia

The youths asserted that *the Frank Attla Program is good for Huslia because it keeps our culture alive*. Youths understand their culture as access to and active participation with all of creator’s decedents. A middle-school girl shared:

*The Frank Attla Youth and Sled Dog Care—Mushing Program means a lot to me. It is a program where the students can participate and have fun with dogs, dog care, and mushing. A lot of fun activities and projects. This program means a lot to Huslia.*

The youths recognize that their upbringing with their culture, around sled dogs, is important because it equips them with skills that will ultimately benefit them in their future. A middle-school girl shared: *It means keeping dog mushing alive and teaching kids life lessons with dogs. Making an impact on a kid’s life.*

In the focus groups, adults expanded on the program’s goal to foster youths’ success and resilience through positive role models who persevered through challenges:

*I think just by using dogs, as an example, we could do that, we could show them that, it’s scary to go to Fairbanks or Anchorage and race, that’s not easy. Or Iditarod. But when we do things like that, we grow from pretty strongly because of it. That is what I want our kids to see, and they’ll do the same thing, you know. Apples don’t fall too far from the tree, but we do is for our kids is going to do. And if we hold back, they are going to hold back too. So we have got to challenge ourselves to push that envelope, and that is what we have to do if we want our kids, and our grandkids, the future generations to be successful.*

#### 3.2.6. A-CHILL Promotes Unity between the School and Community

The youths also shared how the program brought the community and school together for a common goal around cultural pride. A high-school girl shared: *It involves dogs and dog mushing and that’s what I really love. It also means a lot that the mushers and school are working together to make this happen.*

Our student researcher, former FAYSDP participant, and now teacher, Joe, highlighted the impact of incorporating the FAYSDP into school curriculum by promoting diversity and respect between students and educators:

*When it *[FAYSDP]* first started, I was a senior in high school, and I remember before that like in middle school and everything there was a high turnover rate, teachers are in and out. So, therefore, like students don’t really respect the teachers as much, like they’re probably going to leave anyway. We had a teacher leave after 1 week before. So, we don’t have, we don’t really respect them. And they’re from somewhere far away, they don’t really know how to connect with the students. So, after the program started, I could kind of notice that once the teacher started showing interest in our culture, and they had a vehicle to connect the school content with the students; then, the students were able to respect the teachers more, too. And I remember as a student seeing my classmates respect Ms. B more, or something like that, because we had that relationship in class.*

### 3.3. Human Relationships

Youths expressed gratitude and shared the ways the program fostered positive intergenerational relationships, and positive feelings about oneself, one’s culture, and one’s future. Adults expanded on these impacts, contextualized through their observations of change.

Many of the youths discussed George and the creation of the FAYSDP in their digital stories. Youths shared memories of visiting his dogs, listening to his stories, and reflecting on how much he loved and cared for them. A middle-school girl shared: *Late grandpa George has taught and loved us so much. It is speechless how much he has done for our town. I hope that we all continue dog mushing in the future and help each other in these programs.*

#### 3.3.1. Intergenerational Relationships

The FAYSDP stimulates peer-to-peer relationships, youth-to-adult relationships, and importantly, youth-to-Elder relationships. Peer relationships are discussed in Section 3.1.3. The youths highlighted that the common interest in sled dogs deepened their relationship with their grandparents and other local Elders. Relationships with Elders are especially important, as Elders are vital Indigenous Knowledge holders. A middle-school boy reflected on racing his grandpa’s dogs:

*I watch other people race to and this year race I came in first place, I made sure to thank my grandpa since I used his dogs. It felt great to hear that I got first when I crossed the finish line.*

Many community members expanded on the ways dog mushing created authentic relationships between youths and their peers, adults, and Elders. These engagements promoted youths’ interpersonal skills for communicating and active listening. A focus group participant shared:

*I work with youth, and a lot of them are shy. They don’t, wouldn’t naturally just open up to people, or if you’re walking by them, they’ll kind of just, the shy ones will just look down, no eye contact, and they’ll just walk by as fast as they can. But I feel like especially the shy ones they get good relationships with the dogs first. And then like J was talking about, then they’re, pretty soon they’re working together [with their peers], and doing these things that they don’t even realize they’re doing. And talking to the mushers, and they’re all asking questions, and just like what B was saying they have something in common to talk about. And so I think it, for the community it created a, you know, where there was a gap probably before it brought them closer together.*

Amongst the comfort of their friends and especially sled dogs, the students were better able to develop personal relationships with Elders, thus giving Elders more opportunities to transmit cultural knowledge through stories and visits. The youths noted that they learned about how their grandparents, other local Elders, and even ancestors lived. Youths understood that Elders’ stories hold lived experience in facing challenges, and the lessons could be useful later in their lives. In reflecting on photos from the photovoice session, a student wrote: *This program has really taught me many things, with Wilson’s stories and Uncle Floyd and Wilson’s guidance can really teach us great lessons on right and wrong.*

A high-school girl shared:

*Dogs helped me learn more about my culture, with respect and how we used to rely on them. We used them for travelling, for hunting, trapping, bringing medicine and mail from village to village, and just to have them there with you.*

By having active experience with sled dogs and on the land, youths are able to apply Elders’ stories to their own lived experience. A middle-school girl shared:

*My favorite part of being here is the dog program, I like to get out and help out with dog yards. It is a great opportunity for me to be able to learn how our elders lived years ago and how to take care of and raise dogs.*

As youths deepen intergenerational relationships, they look up to their mentors and subconsciously pay close attention to the nuanced details of their mentors’ work. Because youths develop skills to consider and learn things from different perspectives, they can apply those skills to learning without verbal instructions. A community member shared:

*When we are at a Culture Camp and they were doing gang lines and stuff, and the kids were sitting here doing these measurements and stuff, and they’re just, they’re doing it by watching the Elder teaching them. And they don’t even realize it, but they learn a lot, and they learn how to do the measurements and different ways of measuring. And the skills of how to problem solve and everything, they might not mention it, but if you actually watch them do it, I mean I didn’t even know. When you are watching them do that, you can see it. That is one of the things that I got out of the videos and stuff, you could see the change in them.*

#### 3.3.2. Positive Feelings toward Themselves

Reflective of their Koyukon worldview, youths use humor in a number of occurrences often to alleviate stress through laughter: *My grandpa won second place in Iditarod. I was proud of him seeing him win, but I didn’t see him win because I wasn’t born yet.*

In another instance, a middle-school girl elicited humor and humility in her digital story. Her story showed an image of her running sled dogs, with which she included: *By the way, I didn’t fall off that sled. I did before but not that time.*

Youths described how FAYSDP helped them “*be the best me I can be*”, feelings of appreciation for the program fostering healthy relationships, and how sled dogs helped them remember to stay healthy. One high-school boy described positive aspirations toward the future: *With the A-CHILL program, I have been growing exponentially toward success and a better future.*

Some youths also self-identified that having a dog yard will help in their future; a middle-school boy shared:

*Overall, it’s a great thing having a dog yard. It will help my future in many different ways, it teaches me what determination is, it will help me have a better attitude towards work and have a good work ethic.*

Some community members perceived youths’ positive thinking toward their self as a lived embodiment of George’s legacy. In response to how dog mushing helps youths work toward goals and the future, a community member responded:

*I think when Uncle George first started that, those kids went a long way. He instilled in them that they have to love and respect each other. They don’t realize it, but when they are working, they are working in teamwork, and they do a lot of that. And he told them they would go a long way if they respect people and love them.*

### 3.4. Inseparable Spheres

As stated throughout this paper, Indigenous ways of being and knowing are inseparable. Even with modern conveniences, youths still crave cultural events that bring all of creation (human and nonhuman) together and they recognize that these events illuminate their sense of self. A high-school girl expanded on the value of maintaining cultural traditions in the face of modern life:

*I especially love how we are still doing this, sure we now have snow-gos and boats, and cell phone service, but we still have races, KRC’s* [Koyukuk River Championship—a prominent sprint sled dog race happening each year in alternating Koyukuk River communities, Huslia, Hughes, and Allakaket], *potlatches, and cover dishes, things that keep our culture alive.*

The following quotes highlight the interconnectedness that people, place, and dogs have in creating Huslia youths’ sense of self, and how their sense of self and sense of place are one and the same:

*Most of us grew up racing throughout the years. The sound the bottom of the sled makes when we run, that makes me content. At age 13, some of us know how to take care of dogs, run them, and accompany them. Our elders and locals teach us, provide us, and most of all they are our companions.*

A high-school girl shared:

*I love running dogs and racing in competitions. During competitions, there is often a ton on your mind, and it is easy to feel anxious. Even with all these feeling, I love how dogs have almost a magical power to give you a calming peace. While running dogs, I find clarity; it’s a period of time without noise and voices.*

Adults characterized the year-round care of sled dogs as a commitment that promotes life skills, like versatility, resiliency, outcome-oriented decision making, and applying resources (or stories) to daily life. A community member shared the following:

*I notice a lot of the kids here in this community are resilient. They’re able to understand that… there’s a process, and understand that process, and taking it back with them allows them to be a little bit more versatile when they go on and be independent. But just understand that there’s a process, and that goes with the collecting the fish, the food, and how to treat your dogs. I was just saying that I notice that it allows them to be really resilient, they’re able to take the resources that they have, and they’re able to excel from it…It allows them to give them that … edge… they’re really able and understand that it’s a lot, also not along with just that resiliency but unity.*

In concluding the adult focus group, participants reflected on how one of George’s main driving forces behind establishing the FAYSDP was to lower suicide rates. One community member shared this:

*It comes to two things… It’s a sense of purpose, and I heard it in here before, and self-pride. And if you have those things, you know, if you have a sense purpose. I mean within this community…And when you have these things…it builds, other things build around it, and maybe you’re less likely to hurt yourself or what not if you feel like you belong. And that comes with self-pride and sense of purpose.*

## 4. Discussion

### 4.1. Interpretations and Connections

The FAYSDP youths identify themselves as being from Huslia and regard it as their personal identity. Through this assertion, they define Huslia as the landscapes surrounding it and grounded in dog mushing culture. Youths distinguish the physical place of Huslia as more than the material land; they also see Huslia as a group of people who orient themselves around a culture of dog mushing. Therefore, by including being from and of Huslia, youths inherently accept that their sense of themselves is grounded in their sense of place. Through the program, youths build relationships with dogs, people, and the land, live out their culture, and learn Indigenous knowledges for coping, persevering, and working hard through challenges.

The FAYSDP helps give life to these relationships, but also depends on them to exist. Through the FAYSDP, sled dogs engage youths broadly, and Huslia youths appear to have a cultural affinity for sled dogs. In fact, sled dogs are culturally captivating for Koyukon people of all ages and across several communities. Traditionally, before villages were established and families lived a nomadic lifestyle to sustain themselves off the land, people would gather at mid-winter and in spring to celebrate having made it through another harsh winter. Gatherings were essential for sharing knowledge and emotional support through festivities that always involved sled dogs. Many Elders consider these events to be the roots of modern day, renowned champion sprint sled dog races such as the Fur Rendezvous and the Open North American in Anchorage and Fairbanks, Alaska, respectively. The tradition has been a continuous thread in the lives of those making home in the Koyukuk River villages of Allakaket, Huslia and Hughes. Kawagley stressed that teaching and learning is an integral part of Alaska Native cultures, and much of their knowledge is transmitted through social activities [8]. For that reason, a majority of the children have rich experience with sled dog traditions. The sustained tradition of mid-winter and spring gatherings (known today as carnivals), which include dog-races, stood out to youths as an important activity to bond generations and communities. Such bonding and bridging build personal and social resources important for health and wellness [19].

Sled dogs made youths feel welcomed, accepted, and of value. This connects with a well-known quote from the late George Attla, “Dogs accept you as you are” [3] illustrating how dogs can support a child’s positive mindset, confidence, and acceptance of themselves and others. Sled dogs boost youths’ self-esteem by demonstrating inclusivity and compassion. Similarly, an increase in self-esteem was also reported among children and teenagers with cancer after their participation in an intervention including a dogsledding expedition [58]. This translates into teamwork with their peers, as well as intergenerational relationships.

Youths regard sled dogs as all having “their own personalities”. This sense of personhood is activated by their keen interest in sled dogs, but also reflective of their worldview orienting them around kinship and *Kk’adonts’idnee* [59]. Recognizing personhood in nonhumans suggests that the youths respect dogs (and likely other nonhumans) as having their own agency, consciousness, and ability to make decisions. Youths appear to naturally relate to sled dogs and have a natural receptivity for them, thus suggesting that youths are adept to learning from a sled dog’s perspective. Social learning through observation fosters adaptive behavior, which is consistent with *Kk’adonts’idnee* stories and also advantageous as it is less costly to learn best practices from others than through individual learning [60]. As youths further develop their ability to consider and learn from various perspectives, they instinctively apply these skills to people. The FAYSDP creates ample opportunity for youths to engage and develop peer-to-peer relationships. Peer relationships are important in youth development as they lay the groundwork for communication, cooperation, and teamwork skills [61]. Positive peer relationships also promote school connectedness, as well as psychological wellbeing [62]. The program provides space for youths to work together, as well as work with Elders and community members.

Youths and Elders seem to naturally form close relationships over time. Because of youths’ inherent respect for Elders and their knowledge and wisdom, the youths intrinsically observe and imitate Elder and adult role models’ attitudes and actions, specific ways of doing things, behaviors, etc. Over time and with countless exposure, youths bank these various approaches to accomplishing a singular objective, largely without verbal instruction. This style of learning rings through FAYSDP, engaging youths to learn through attuned observation, be sensitive and attentive to Elders/role models, and creatively apply what they learn. The culturally shaped observational learning approaches that Indigenous children come to know and practice allow them to figure out their world and figure out solutions to life’s problems [63]. Educational scholars, as well as youths themselves, have identified problem solving and critical thinking skills as important skills for future success in a changing world faced with new challenges [64,65]. Culturally tailored life-skill interventions that build problem-solving skills can also facilitate hope and reduce suicide risk [66]. Traditionally, Indigenous adults and Elders practiced intentional “non-interference” so as to not insert their perspective on impressionable young minds [67] (p. 80). Instead, adults and Elders showed children how to engage with all creations through cultural practices of survival to develop their critical thinking skills. Simultaneously, Elders used stories as a tool for guidance. Youths would reconsider these stories and lessons repeatedly over time through new experiences, observations, and responsibilities to help shepherd their lives and overcome challenges.

The FAYSDP fostered and deepened youth relationships with adults and Elders. Such intergenerational connections promote resilience for Indigenous youths and are protective against suicide risks [68,69]. Youths recognized that Elders’ stories were meaningful because they held tried and true lessons for life. Indigenous knowledge relies on Elders’ transmission of personal and traditional stories because stories were (and still are) a tool for guidance and promotion of critical thinking skills such as observation, attention to details, and problem solving. Youths recognized that participating with sled dogs and/or being on the land was a “great opportunity” for them to critically consider and apply Elders’ stories. The FAYSDP and modern youths are especially reliant on Elders because youths today are growing up in a time starkly different than that of their grandparents, and supportive relationships are critical for navigating the challenges they face in their transition to adulthood [70]. Accepting and valuing diversity is also vital for Indigenous knowledge transmission. Diversity promotes accepting differences, adapting to change, careful action, and consideration about the future.

Youths’ digital stories and photos highlight their attunement to the land. Youth and adult narratives underscore the sense of peace that youths attain from running sled dogs. They also underscore taking in the land, “watch[ing] the birds and look[ing] at the trees and other stuff”, as contributing to their peace. In being on the land, youths are creating a foundational understanding of the intricate relationships among the landscape, animals, and people and relational interplay therein. This finding confirms the value of nature-based interventions in Indigenous and non-Indigenous settings reported previously [71,72]. This environmental appraisal and analysis of ecological processes help youths recognize the “creative force flowing in and around them at all times”, promoting their honor and reverence of nature [8] (p. 89).

While humility is an Athabascan cultural value intended to keep Koyukon people from inserting themselves into a hierarchy, superior to other humans and nonhuman creation, it seems that lessons on purposeful feelings toward oneself are still transmitted. Youths’ narrations illustrated positive feelings towards themselves and attitudes of “never give[ing] up” and believing “you can achieve when you can put your shoulders into it”, collectively suggesting intentionality for their future. The youths’ stories demonstrate agency, which is a critical part of the growth mindset that emphasizes working hard to face and overcome challenges [73,74]. Feelings of agency and mastery are interwoven with community connections in Indigenous communities; relationships build confidence to solve problems with support of important others [75]. Because youths identify themselves as grounded in relationships with their people, the land, and animals, the community’s involvement and the embrace of the FAYSDP reinforce the youths’ positive aspirations. The youths’ stories also illustrated the ways in which FAYSDP fostered connections to culture and self. A clear sense of cultural identity facilitates a strong sense of self, promoting psychological wellbeing and feelings of self-worth [76,77].

The youths also valued the way the FAYSDP unites the school and community together. A multicultural approach to teaching and learning promotes cultural diversity by bolstering cultural equity for all individuals and groups [78]. Cultural diversity discourages “stereotypes, monocultural instructional methodologies, ignorance, social distance, biased research, and discrimination and prejudice” [78] (p. 26). Advancing cultural diversity through multiculturalism contends that education starts with parents and caregivers as primary educators. For this reason, the school curriculum should acknowledge education from home and the community; otherwise, “this lack of continuity and recognition of cultural and indigenous knowledge is a confirmation that the curriculum enforces and exposes learners to Western knowledge only, at the expense of local knowledge; thus, it emphasises colonisation of education system from an early age of schooling” [78] (p. 36).

Lessons taught underneath the Indigenous knowledge domain highlight a cultural conditioned astuteness, reverence, and reciprocity for relationships with human and nonhuman kin. These lessons funnel down to construct a worldview that vitalize survival and stimulate creative problem solving to persevere despite peril or hardship. To actualize and utilize Indigenous worldview, one must have kinship-like concern for these relationships and an inherent receptivity to them. The FAYSDP appears to be a platform that strengthens customary relationships through active engagement with sled dogs, the land, and the community. The FAYSDP is a framework gives all youths access, time, and space to establish and develop a strong sense of place and self. This sense of place and self is interconnected and holistic, consistent with traditional ecological knowledge: “The self is defined in relation to one’s tribe and family, as well as one’s land or place; all are intrinsically connected, and one cannot understand oneself outside of those relationships. Those features are not accidental but essential to who one is, creating a strong ‘sense of place’ in Indigenous communities” [4] (p. 116). The FAYSDP framework seems to enable youths at all phases of cultural awareness and attunement to foster and strengthen traditional relationships with animals, the land, and community, while simultaneously teaching them life lessons.

Reflective in both youth and adult focus group accounts, sled dogs are a time-tested cultural approach to teaching, learning, and accessing lessons for life and Indigenous knowledge. Even in a time vastly different than that of their grandparents and with modern conveniences at their disposal, sled dogs are a year-round commitment that demand engagement, premeditation, and intentionality by way of socializing with the dogs and meeting their needs with water, fish, and wood to cook their daily meals. Kawagley and Barnhardt wrote:

*Being in and with the environment the whole year round, students can experience the vicissitudes of seasons, flora, fauna, sunlight, freezing, thawing, wind, weather permutations, gaining intimate knowledge about place using their five senses and intuition to learn about themselves and the world around them*

[79] (pp. 14–15)

The perspectives that Huslia youth and adults shared illustrate how FAYSDP fosters sense of place within the community and promotes wellness and identity through teaching cultural life skills and values. Through the program, youths build relationships with dogs, people, and the land, live out their culture, and learn Indigenous knowledges for coping, persevering, and working hard through challenges. Much of this learning is achieved on the land by working with dogs and overcoming obstacles. Through this active engagement, youths also come to understand who they are in relation to others and their place.

### 4.2. Strengths, Limitations, and Implications for the Future

Our study helps shed light on the ways a culturally grounded, community-led, and school-integrated program affects wellbeing, from the perspective of an Indigenous framework centered on the interrelationships among humans, animals, and the environment, which were all intricately interconnected in the youths’ stories.

The holistic nature of the findings lend a hand to several implications; this Special Issue likely portrays many related examples thereof. Importantly, it is my hope that these multifaceted findings and the insight of a Koyukon (or Indigenous) worldview urge researchers to commit to dialog within their research teams, especially those partnering with Indigenous groups. Dialogue should center around challenging implicit assumptions suggesting that Western knowledge is superior to Indigenous knowledge, and it should recognize that all research and researchers are shaped by their worldviews and their experiences, that language reflects (even implicit) biases, and that a multitude of worldviews influence our work. *Etuaptmumk* (Mi’kmaw for Two-Eyed Seeing) inherently recognizes diverse worldviews and ways of knowing, firmly opposes the common place notion suggesting the integration of Indigenous into Western knowledge, and has been useful across a variety of academic disciplines [80]. Two-Eyed Seeing acknowledges that Indigenous and Western sciences should be applied in parallel, taking the strengths of both systems for a clearer and broader understanding. Unique to this approach, Two-Eyed Seeing is centered on ethical obligation and responsibility to uphold the integrity of the knowledge and where it emerges. While there are many examples of Two-Eyed Seeing in practice, diverse research teams vested in promoting the ethical underpinnings of Two-Eyed Seeing may benefit from the First Alaskans Institute dialogue agreements [81]. The agreements are led by Indigenous principles and values but help guide critical conversations, with respect, and they can be applied to many groups.

This study helps give voice to Indigenous people’s viewpoints on their interconnected sense of place reinforced by a specific culturally based program. Teachers and community members have previously noted improvements from the FAYSDP in student behavior and outcomes, and positive community transformation [82]. The current study supports and expands on this work by integrating culturally resonant narratives from youth and community members that express how culturally based active engagement facilitates a sense of place and purpose, perseverance, and wellness. While this study does not contain prepost quantitative assessments of outcomes such as sense of purpose and pride, it does distill Koyukon ways of perceiving themselves and triangulate youth voices with adult reflections. This contributes to validity through the integration of multiple perspectives and the utilization of community dissemination to deepen understandings by engaging community expertise [57,83].

Future work should include collaborations with Indigenous community members, to build youths’ sense of place into culturally specific intervention components promoting wellbeing. A clear message of the Alaska Native students’ digital stories was their natural interest and trust in Elders as teachers. Entirely supported by the community, this cultural value and strength is significant in fostering social and natural connections to promote youth wellness. Although sled dogs and their rich history gave structure to the FAYSDP and A-CHILL, their foundation was the incorporation of Elder teachers as part of Western education instead of separate from it. Along with intergenerational community member support of Elders, with an inherent strong love of and dedication to youth, future program options with or without sled dogs involving Elders are limitless in any Alaska community. As respected teachers in daily Western education, Elders’ impact on youth and community is profound. The implications of studies like this one can only positively support youths feeling stress in facing and adapting to living traditional ways in a fast changing modern world.

The study also highlights the impact of integrating Alaska Native ways of teaching and learning. Creating a space where Elders are recognized as teachers whose stories and lessons help the youths connect the past to the present and eventual future is an important step, and it includes learning that values observation, experience, and reflection over lectures or direct verbal instruction. The land as the classroom offers a living textbook where relationships between humans, animals, and the environment can be connected, observed, and experienced. Further research can be conducted to explore the impact on retention and graduation rates of students participating in programs such as FAYSDP.

## 5. Conclusions and First Author’s Reflections

### Janessa Newman

Alas, we circle back to a final point in story. One might argue that we never left a story; the theoretical underpinnings and the vast web of relationships they give life to naturally adhere to reoccurring and cyclical patterns. Throughout the course of writing this publication, learning from Indigenous authors, and reflecting on my own experience, much of this knowledge was revealed to me. These were not inherent words and passages I was raised with, but the emphasis on relationships rang very clear throughout my life. Through acts of colonialism and assimilation, much of our knowledge has been “put to sleep”, and that is not to say that it is gone, but to say it will come back. These relationships, storied by children are living proof of that. So much of what was discussed in this paper can only be understood within context, taking many complex and interconnected relationships into consideration. It may take reading several times over to grasp even half an understanding, but that is why stories are so powerful. There are many ways to interpret stories; there is no instant gratification and no one lesson, but many that can only be realized over the course of learning and experiences throughout life.

There is so much that we, as Indigenous people, have to say in our hearts that simply do not translate into English. Stories, analogies, and metaphors can sometimes feel like the only ways we can give life to what our hearts have to say. Not only do we know this, but Western researchers also recognize this; it is why digital stories are effective. The following is an excerpt from the adult focus group, and a quote I revisited numerous times throughout writing this paper:

*Ultimately I think dog mushing has done something for our people that… I don’t think people really recognize it. What I mean by that is look back in our history, and we see a lot of champions come from Koyukuk River, and the challenges, and what it took for them to take that challenge, to push the envelope, get outside of their comfort zone. I think that is really important. Especially… in light of how our lives are today… It’s just a new diameter… But it is really important for our kids to go on and achieve goals and education so they can use that to set themselves up for their future, create a foundation for themselves… They [Elders] had the will and the drive…But I think that’s one thing I never see discussed in the Frank Attla Youth Program or Huslia, or any other, what it takes for an individual to get out of their comfort zone, and go out into the world somewhere and try to achieve a goal. I think that is really… we know that is important… I look at our education program and there is nothing wrong with our people living in our villages, there is nothing wrong with that at all. The only thing about it is it is difficult to make a living; so, our young people have to have a means of some kind of tool that they could support themselves… In the last 15 years, a lot of our students leave our schools, and this school included. And they go and they run into difficult times in trying to get their college degree or whatever. They eventually come home, which is fine, there is nothing wrong with that. But it is my hope and my dream to get a higher percentage of our school (students) just continue on, and tough it out, and get their degrees, so they can use that as a tool to support themselves, but ultimately, to make society a better place than what they find. For them to come back and help in our community, where help is really needed. That is what I think this was designed to do and I hope that’s how it’s interpreted someday… But I don’t think I have heard enough of how these students are that learn how to set goals for themselves and stay on the task, stay the course and achieve their goals. So ultimately, they have turned around they’d be able to use that to better themselves, better their lives. To be able to support themselves, that is the key right there. That is what I really want to see for our young people because man, like I said earlier, the dynamics are changing. Our old way of life of living on the land, it is virtually nonexistent now… So that is why it is really important for our young people to get a degree in college, or military service, or GTE, or some way to put tools in their toolbox where they can use it to make a living. I think, I know that is part of why George started A-CHILL. He saw that, and that is what he told me.*

This passage actually inspired the interpretation of the research. It took reading several times over, with consideration of the Indigenous researchers who inform this work, and recalling my own experiences to gradually understand and recognize the inherent value within. Essentially, this community member provides a storied account for Elders’ upbringing with the land and in relation to others as imperative for understanding and transferring Indigenous knowledge. The Koyukon people, like all Indigenous peoples, have cultivated a way of life that has empowered them to persevere against stark odds. They refined a worldview that allowed them to thrive in the face of adversity. Indigenous knowledges foster an obligation of doing what is “right” despite hardship and obstacles.

In true retrospect of my Koyukon worldview, I hesitated to draw findings, suggesting them as final results. My interpretation of this paper as a whole is the result of my knowledge and my experience thus far. Others might interpret it entirely differently, and even 20 years from now I might read this with entirely new insights. I simply hope that I did Huslia justice.

All this is to say and draw an example of how much power Indigenous voices hold if we can take the time truly listen, pause, and internalize all they are trying to share with us. We cannot strip their words from their lived context, in relation with people, animals, land, and water, simply because English is oriented around nouns. These stories, words, and knowledge belong to the Huslia community, the land that encompasses all landscapes and animals therein, and their ancestors.

The skills highlighted in these Koyukon children’s stories are only the tip of the iceberg. These skills have empowered Koyukon (and other Indigenous) peoples to survive through times of ancient peril and persist through modern life’s obstacles and systemic policies intended for their demise. The Indigenous worldview, as well as the knowledge system within, makes it possible for lessons and skills transmission to adapt, and George knew this.

In the end, George Attla left his community a gift. A gift born out of pain—illness, boarding school, loss of culture and knowledge, and ultimately the loss of too many Koyukon and Indigenous children alike. Although his dog mushing success brought him fame, success did not heal him; the culture and the dogs did. In his later years, he was able to reflect and realize the dynamic power his culture provided and that the dogs were the conduit through which he could help the youth and his community. He understood, very clearly, that bringing together the people, on the land, through sled dogs was sure success. The history books might cite the fact that George was a Huslia Hustler and the winningest musher, but the hearts of the people involved with the Frank Attla Youth Sled Dog Care—Mushing Program carry the difference he made in their lives. In the Raven unveiling itself to me and the resulting energy it carried, coincidentally, on the anniversary of George’s passing are proof that, even in death, nothing in life is final.

## Figures and Tables

**Figure A1:**
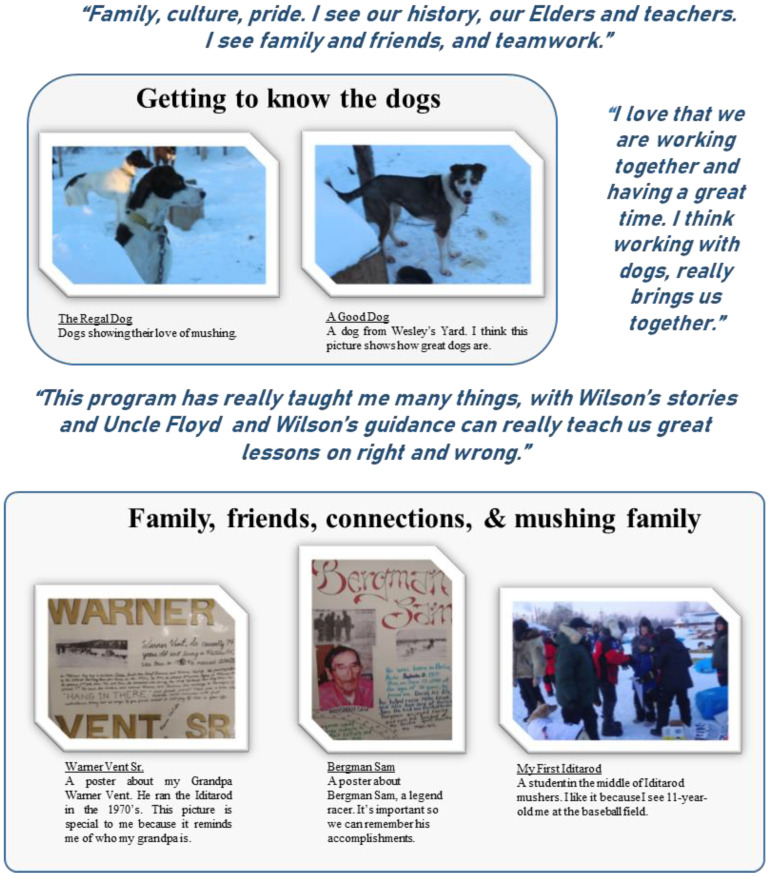
Photovoice themes “Getting to know the dogs” and “Family, friends, connections & mushing family”. The text under each picture is the title and description written by the youth participant to describe their picture. The blue emphasized quotes around the themes correspond to comments posted by youth participants upon discussion.

**Figure A2:**
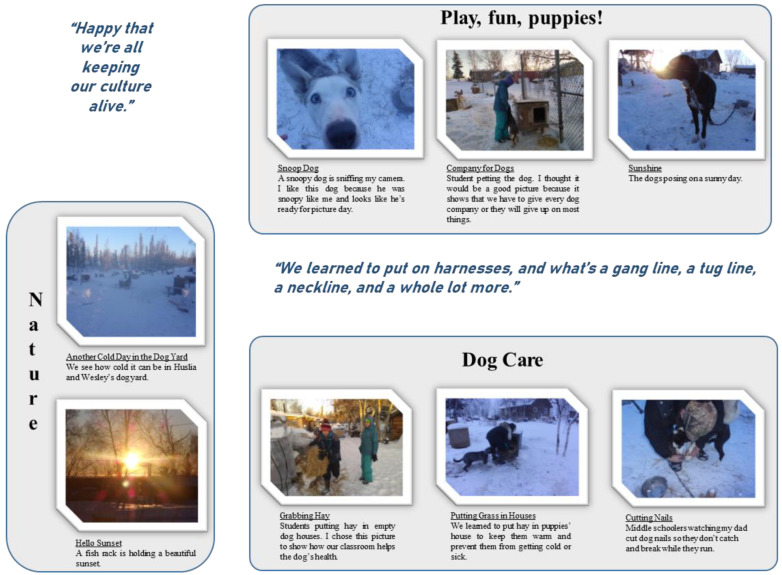
Photovoice themes “Play fun puppies”, “Nature” and “Dog care”, along with youth participant descriptions of their photos, and youth quotes.

## Data Availability

The digital stories that youths and facilitators created can be seen at https://www.achill.life/what-we-did (accessed on 30 September 2022), and the photovoice exhibits can be seen at https://www.achill.life/what-we-learned (accessed on 30 September 2022). Data (including quotes) are also contained in the current article. Additional data including the codebook organized around elements of social and cultural capital and cultural values are available in a previously published manuscript based on the project [19]. The full adult focus group data are not publicly available due to considerations of confidentiality and community ownership, but themes and quotes are contained in the current manuscript.

## Appendix A

Photovoice themes with examples of photos are shown in Figure A1 and Figure A2.
